# St. Maur; an Earl's Wooing

**Published:** 1879-11

**Authors:** 


*St. Maur; An Earl's Wooing.*—By John Carroll, Caves Baltimore Co., Maryland. Publishers: T. B. Peterson & Bros., Philadelphia, 1879.

This is one of the series termed "New Society Novels," and deserves a prominent place in the standard literature of the day.

It contains nothing objectionable or common-place, and is highly creditable to its author, who is a well known and highly esteemed citizen of Maryland. The scene is in England, though some American characters, drawn without exaggeration, figure in it. It is decidedly sensational, with a well-constructed story, which might be regarded as too highly wrought, were it not that every mystery is set even at the close. The various action takes place in high, middle, and what may be called low life—though it does not go so far into the depths of the latter as "Oliver Twist." One of the best characters, of whom too much is not made, is a clever Detective. Most of the scenes are worked out with great effect; and the destruction of a great country mansion, by fire, in England, in which the heroine and hero are saved by the devoted self-sacrifice of Trevellyan, the Earl's tried and true friend, is most powerfully written, and the death scene of the latter, is a wonderful bit of tender pathos. There is an exquisite little poem in it. The story has numerous good points, and is nicely told. The author is well acquainted with London society, and in all respects this romance of the present time will be found highly original. St. Maur; an Earl's Wooing, is published in a large square duodecimo volume, paper cover, price 75 cents, uniform with "L'Assommoir," and will be found for sale by all Booksellers and News Agents, and on all Railroad Trains; or copies of it will be sent to any one, to any place, at once, on their remitting 75 cents in a letter to the Publishers.

---

